# Mobile App Rating Scale for Health Care Professionals to Assess the Quality of mHealth Apps: Questionnaire Development and Psychometric Analysis

**DOI:** 10.2196/48828

**Published:** 2026-07-31

**Authors:** Albie Sharpe, Yiong Huak Chan, Amarasinghe Arachchige Don Nalin Samandika Saparamadu

**Affiliations:** 1 Discipline of Public Health, School of Human Performance, Rehabilitation and Population Health, University of Technology Sydney, Sydney, NSW, Australia; 2 Biostatistics Unit, Yong Loo Lin School of Medicine, National University of Singapore, Kent Ridge, Singapore, Singapore; 3 Department of International Health, Johns Hopkins Bloomberg School of Public Health, Johns Hopkins University, 615 N Wolfe St, Baltimore, MD, 21205, United States, 1 4109553543, 1 4105029809; 4 Department of Nutrition and Food Studies, College of Public Health, George Mason University, Fairfax, VA, United States

**Keywords:** Mobile App Rating Scale for health care professionals, MARS for health care professionals, pMARS, psychometric analysis, structural equation modeling, item response theory, scale development, internal consistency reliability, eHealth, mobile health, mHealth

## Abstract

**Background:**

Many frameworks and tools are available to evaluate the quality of mobile health apps (MHAs), which are increasingly used by health care professionals (HCPs) for accessing medical information, clinical decision support, and communication. However, existing tools are not well equipped to assess the quality of apps designed for HCPs from their perspectives.

**Objective:**

We aimed to develop a new tool based on the Mobile App Rating Scale (MARS) to capture the unique perspectives of HCPs on MHAs. We then conducted a psychometric analysis of this new questionnaire to determine its effectiveness in assessing the quality of MHAs designed specifically for HCPs from their perspectives.

**Methods:**

This study was conducted in 2 phases. In phase 1, the original MARS tool was adapted for HCPs through expert panel review and subsequent qualitative interviews, resulting in the development of the pMARS (MARS for health care professionals) tool. This phase focused on establishing face and content validity. Qualitative interviews were conducted with HCPs from a tertiary hospital in Singapore to gather their perspectives on the tool’s structure, clarity, applicability, and usability. In phase 2, we invited HCP participants to complete pMARS based on their experience with the LabMed app, an mHealth tool designed to provide medical laboratory–related information to HCPs. We established the construct validity of pMARS through multiple psychometric techniques. Internal consistency reliability was measured using the Cronbach α, while structural equation modeling was used to examine the interrelationships among latent constructs. Additionally, we used item response theory (IRT) to evaluate each item’s impact on latent constructs of interest, that is, discriminative performance of individual items within each domain.

**Results:**

Based on the results from phase 1, the pMARS comprised 26 items across 5 domains: engagement, functionality, aesthetics, information, and subjective quality, refined through interviews with 10 HCPs. In phase 2 (n=218), pMARS demonstrated good internal consistency reliability across all domains (Cronbach α=0.855-0.931). Structural equation modeling demonstrated that functionality had the strongest influence on end-user willingness to use, recommend, and purchase the MHA (*P*<.001). IRT identified that customization and interactivity of the engagement domain had a weak impact on latent constructs, whereas entertainment had a higher impact. Ease of use and gestural design had a weak impact on the functionality domain, whereas arrangement and size of content and quantity and quality had a strong impact on the aesthetics and information domains, respectively.

**Conclusions:**

This study reports the development and psychometric analysis of pMARS. Our findings demonstrate strong internal consistency reliability and construct validity, supporting its potential use in health care. Further research should validate pMARS across diverse MHAs and contexts and apply IRT to further refine its precision and efficiency.

## Introduction

Mobile health (mHealth) apps (MHAs) offer the potential to deliver accessible, efficient, and cost-effective health care [1,2]. These apps are used not only by patients for health education and self-care but also by health care professionals (HCPs) [3]. HCPs rely on MHAs for many purposes, including consultations between HCPs, intersectoral communication, and access to information for HCPs at the point of health care delivery [3,4]. However, the quality of MHAs designed specifically for HCPs can vary significantly in terms of the accuracy of the information provided, functionality, data visualization effectiveness, credibility, and other key factors. Poor-quality or misleading content can pose serious risks to patient health and safety, underscoring the need for reliable quality assessment measures [1,5-7]. Although several frameworks and tools have been developed to evaluate the quality of MHAs, the Mobile Application Rating Scale (MARS) remains one of the most widely used and recognized tools for this purpose [8-10].

MARS is a multidimensional tool designed for experts to assess the quality of MHAs [10,11]. It consists of 23 questions or items, with 19 of these organized into 4 objective dimensions: engagement (5 items), functionality (4 items), aesthetics (3 items), and information (7 items) [10]. MARS has been widely applied in the evaluation of apps related to health, well-being, health care, and medicine [12-17]. Researchers have validated MARS across multiple languages, demonstrating that its psychometric properties are consistent with those of the original English version [11,17-20].

Given the expertise and training required to administer MARS, a simplified version, known as uMARS (user version of the MARS), has been developed for end users [21]. Items requiring expertise and complex terminology have been removed from uMARS. While uMARS has gained wide acceptance for its utility in assessing the quality of various MHAs [22-25] and has been translated into other languages [26], it has also faced criticism. Specifically, it has been noted that uMARS may inadequately capture end user perspectives on quality, and some have suggested that it should include emerging mHealth technologies to enhance its comprehensiveness [27].

Despite the widespread use of tools for assessing the quality of MHAs, there have been only a few attempts to evaluate these tools from the perspective of HCPs as end users. This evaluation is crucial for several reasons. First, HCPs are reported to have unique requirements when assessing quality, including access to patient data, data security, and evidence-based clinical content [28]. Second, MHAs designed for HCPs must cater to clinical needs and other professional decision-making processes. Unlike MHAs for general users, these apps are also subject to professional and organizational policies that mandate specific security features. Third, HCPs often face demanding schedules, with limited time to thoroughly assess digital tools due to their primary responsibilities in patient care and clinical decision-making [29]. Finally, current assessment tools, such as MARS and uMARS, may not fully meet these specific functional requirements or accommodate the time constraints experienced by HCPs. Consequently, developing a new, specifically designed, and simplified questionnaire that can be administered quickly would effectively address these gaps and provide a more relevant evaluation tool for HCPs as end users.

Moreover, involving HCPs in the development of MHAs is widely recognized as a key factor in creating safe and effective apps [28,30,31]. A tailored assessment tool would enable MHA developers to address the unique priorities of HCPs. In addition, by capturing the distinct perspectives of HCPs using a dedicated tool, service providers can obtain more accurate and practical insights, ensuring that MHAs are suitable for professional use and health care delivery. Therefore, we aimed to (1) develop a new MHA rating scale tailored for HCPs based on the widely validated MARS framework and (2) conduct a psychometric analysis of this new questionnaire to determine its effectiveness in assessing the quality of MHAs designed specifically for HCPs.

This paper describes the development of the new rating scale and evaluates its initial application using the LabMed app, an mHealth tool designed to provide medical laboratory–related information to HCPs [29,32]. We present our Methods, Results, and Discussion sections in accordance with the “recommendations for instrument and scale development and testing” [33,34].

## Methods

In this section, we discuss the specific methods and materials used in the study: (1) the process of adapting MARS for use by HCPs with the goal of establishing judgment validity and the qualitative methods used in this process (phase 1) and (2) the assessment of reliability and construct validity of the new tool, pMARS (MARS for health care professionals), through Cronbach α and structural equation modeling (SEM), respectively, followed by the utility of item response theory (IRT) in identifying weakly impacting or performing items (phase 2).

### Phase 1: Development of the New Rating Scale (pMARS) and Establishment of Judgment Validity

#### Overview

**Figure 1.**
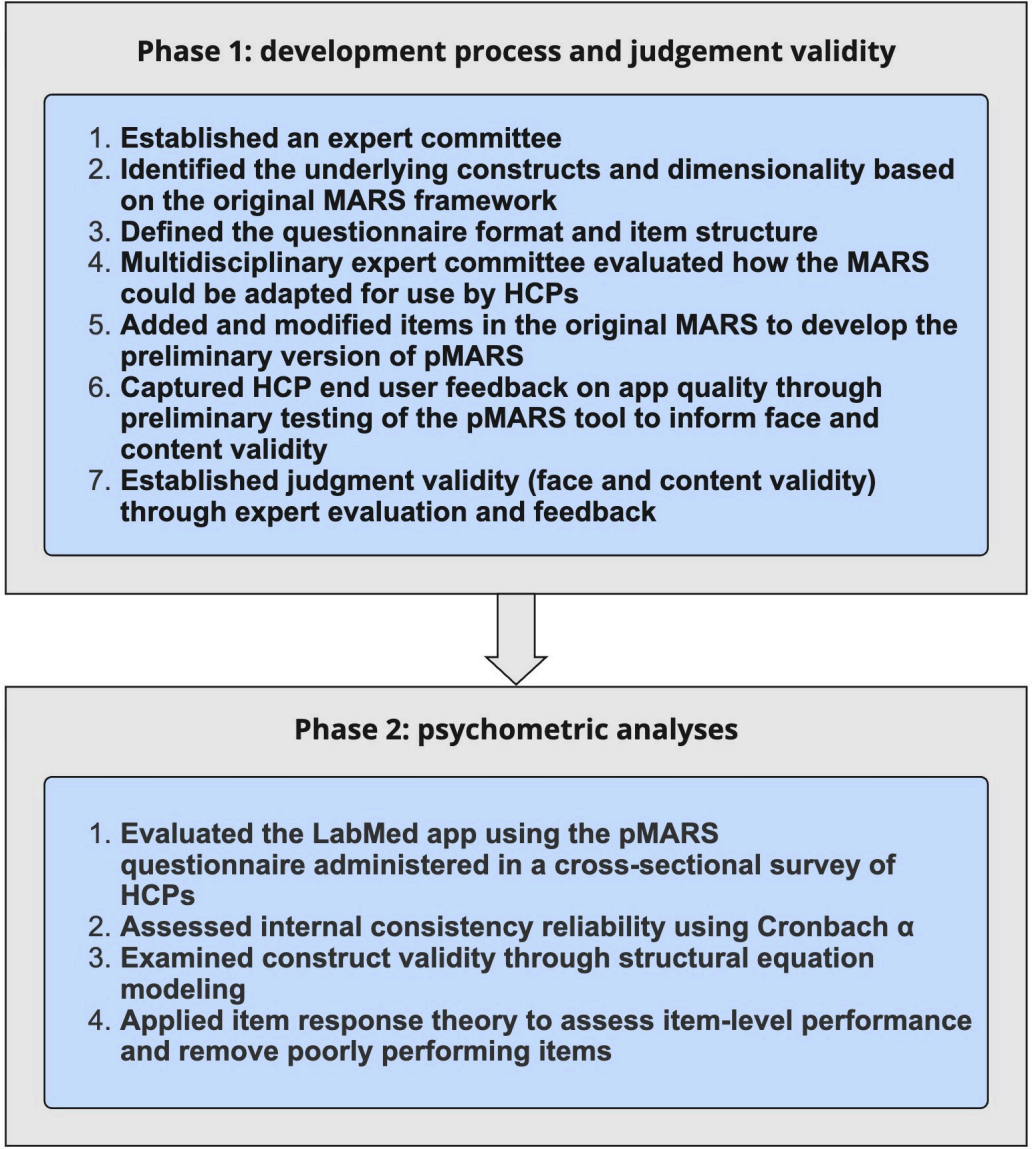
Study outline. HCP: health care professional; MARS: Mobile App Rating Scale. pMARS: Mobile App Rating Scale for health care professionals.

Judgment validity was based on face and content validity [35]. Face validity is the degree to which respondents judge the questionnaire items to be valid. Content validity is the extent to which the observed variables in a questionnaire are representative of the theoretical constructs or latent variables (eg, entertainment, interest, customization, and interactivity) and latent constructs (eg, engagement, functionality, and aesthetics). A study outline is shown in Figure 1.

#### Expert Evaluation

Judgment validity as a whole was approached using the following steps: establishing an expert committee, identifying dimensionality of constructs, determining the questionnaire and item formats, adding items, determining length, reviewing results, and revising [35]. This step was guided by the anticipated clinical, informational, and workflow-related needs of HCPs when evaluating MHAs for professional use.

#### Data Collection

Data collection focused on systematically eliciting expert judgments on the relevance, suitability, clarity, and completeness of MARS items when assessing MHAs intended for HCP use. We assessed content validity with the following criteria: (1) questions were clear and easy to understand, (2) questions covered all the important aspects of each domain, (3) the questionnaire did not omit important questions regarding quality of MHAs, (4) the questionnaire was suitable for replication studies, and (5) there were no violations of privacy. A 3-member panel of multidisciplinary experts comprising a physician, a public health expert, and a human-computer interactions specialist evaluated the relevance and suitability of the original MARS domains and identified items for adaptation to HCP end users.

#### Data Analysis

The aim of this analysis was to identify key domains and constructs from HCP perspectives, eliminate multiconstruct items, ensure appropriate language for HCPs, and review questionnaire length. Feedback was synthesized through iterative review and consensus among the experts to determine item retention, modification, and/or removal. A draft tool with 23 items across 5 conceptually defined domains was developed. Items were not reverse scored. Domains were treated as conceptual groupings rather than independent subscales. Scoring of the tool was carried out by averaging the item scores within domains and across the full pMARS tool.

#### Interviews

To further establish face and content validity from an end-user perspective, qualitative interviews were conducted with HCPs.

##### Data Collection

To assess pMARS’s ability to capture quality and other key characteristics, we recruited a sample of HCPs (n=10) through both convenience and purposive sampling for face-to-face interviews using a semistructured interview guide provided in Multimedia Appendix 1. A convenience sampling method was used to recruit HCPs who had recent contact with the clinical laboratory. A purposive sampling method was used to ensure that both physicians and nurses were represented in the sample. One researcher (AADNSS) was present for a week at the clinical laboratory to recruit participants.

Demographic details, excluding personal identifiers, were collected during the interview. Participants first completed version 1 of the pMARS tool, then were interviewed about their experiences and asked for suggestions for improvement. On average, the entire interview process lasted 25 to 30 minutes and was conducted in a private, quiet space within the hospital.

### Interview Analysis

Audio recordings from the interviews were transcribed verbatim and handwritten notes were compiled without participant identifiers. Anonymized transcripts were analyzed using a deductive coding process [36]. One researcher (AS) conducted the initial coding, categorizing participant feedback based on the role pMARS played in assessing the app. Preliminary codes and emerging themes were discussed by the researcher and a collaborator until consensus was reached. The final coding scheme was subsequently reviewed and validated by a second researcher (AADNSS). Findings from this phase were used to refine item wording and enhance the content validity of the pMARS tool.

### Phase 2: Evaluation of Reliability and Construct Validity of pMARS

#### Overview

To evaluate the pMARS questionnaire, a cross-sectional analysis was conducted with the users of the LabMed app between March and August 2019 at a tertiary care general hospital in Singapore.

#### Data Collection

The physicians, residents, nurse clinicians, and registered nurses in the hospital who used the clinical laboratory services and had experience using the LabMed app were eligible to take part in the study.

We used a simple sequential sampling approach to recruit participants. The questionnaire was administered using 2 parallel approaches. First, a researcher (AADNSS) systematically visited all hospital wards in person and distributed printed questionnaires, which were collected periodically during the data collection period. Second, the questionnaire was made available through the hospital intranet via the REDCap (Research Electronic Data Capture [37]) electronic data capture platform. The HCPs were informed of the online option through a single mass email campaign conducted by the hospital’s marketing department. Response was voluntary.

REDCap is a secure, web-based software platform designed to support data capture for research studies. We aimed for a participant-to-questionnaire-item ratio of 8:1 to 10:1, resulting in an estimated sample size of 184 to 230 [35].

#### Data Analysis

All forms of questionnaires were transcribed into a Microsoft Excel file by 2 researchers working together to minimize transcription errors. STATA (version 17.0; StataCorp) was used for analysis [38]. The Cronbach α was used to show the internal consistency reliability of pMARS: 0.80 to 0.89 was considered good, and >0.90 was considered excellent [39]. A high α may indicate redundancy; however, it is not the sole determinant of item appropriateness [40]. Face and content validity, along with factor structure, were essential in determining the questionnaire’s ability to accurately measure the intended construct.

**Figure 2.**
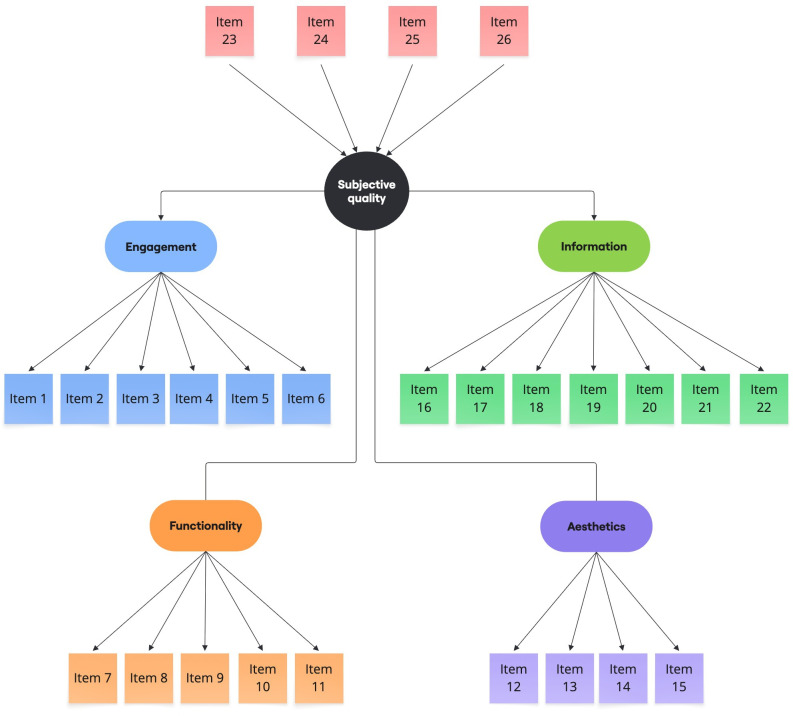
The model used in structural equation modeling.

SEM was used to evaluate the interrelationships between the latent constructs after adjusting for age, sex, and HCPs’ role in the hospital [1]. SEM assumed a latent construct for the observed items of each subscale, and a higher order factor—subjective quality—accounted for correlations between the factors (Figure 2).

Lastly, we used IRT to identify each question’s impact on the latent constructs, that is, engagement, functionality, information, aesthetics, and subjective quality. Unlike classical test theory, which assumes all items contribute equally to the overall score, IRT allows for identifying items with varying discrimination, making it ideal for optimizing and shortening measurement tools without compromising validity and reliability. The primary purpose of using IRT was to refine the questionnaire, resulting in a precise, valid, and relatively brief pMARS that minimized the completion time and response burden [41].

### Ethical Considerations

This research project received an ethics review exemption from the Projects Committee of the Department of Laboratory Medicine at Ng Teng Fong General Hospital, Singapore. The exemption was granted in accordance with the department’s policy for minimal-risk research involving health care staff and was reviewed and approved by the head of the department. All participants were provided with an information sheet describing the study objectives, the voluntary nature of participation, and the commitment to data confidentiality. Informed consent was obtained before participation in each study phase. Participants who completed the qualitative interviews (phase 1) or paper-based questionnaires (phase 2) provided written consent, while those who completed the online survey via REDCap on the hospital intranet platform provided electronic consent.

Basic demographic data, including age, biological sex, and professional role, were collected. No personally identifiable data were collected. All files were stored on password-protected institutional drives accessible only to study investigators. In phase 2, participants were offered a Singapore $10 (S$1=US $0.78 as of June 17, 2026) voucher for a local coffee chain as compensation for their time spent completing the questionnaire. No other payments or incentives were provided.

## Results

Phase 1, the qualitative study, included 10 participants. Phase 2 included 218 participants who responded and returned completed questionnaires. Selected sociodemographic characteristics of the participants are presented in Table 1.

**Table 1.** Demographic characteristics of the participants in phase 1 and phase 2.

Characteristics	Values
Phase 1	
Age (years), mean (SD)	28.4 (8.81)
Sex, n (%)	
Male	3 (30)
Female	7 (70)
Role in hospital, n (%)	
Nurse	8 (80)
Doctor	2 (20)
Phase 2	
Age (years), mean (SD)	30.4 (7.2)
Sex, n (%)	
Male	39 (17.9)
Female	176 (80.7)
Missing data	3 (0)
Role in hospital, n (%)	
Nurse	154 (70.6)
Doctor	47 (21.6)
Missing data	17 (7.8)
Specialty, n (%)	
Surgical specialties	26 (11.9)
Medical specialties (inpatient or acute care)	91 (41.7)
Community hospital	9 (4.1)
Unspecified	92 (42.2)

### Phase 1: Development of pMARS and Assessment of Its Judgment Validity

The first version of the questionnaire consisted of a demographic data section and 5 domains. Certain question stems were reworded and/or split into 2 separate stems to ensure that there were no double-barreled questions, for example, item 1 of section A of MARS reads, “Is the app fun/entertaining to use? Does it use any strategies to increase engagement through entertainment?” This was broken into two separate question stems: (1) “Is the app fun/entertaining to use?” and (2) “Does the app use strategies to increase engagement through entertainment (e.g., strategies such as interactivity/gamification)?” This version of the questionnaire consisted of question stems presented in question form and shortened but descriptive answer stems presented as a Likert scale. Moreover, almost all the question stems had examples or descriptive statements to further elaborate or clarify the ideas.

Insights from the qualitative assessment, categorized into 4 key areas, guided the multidisciplinary panel in developing the second version of pMARS at the end of phase 1 (Table 2).

**Table 2.** Quotes from the qualitative interview participants (n=10) in relation to specific functionalities of the Mobile App Rating Scale for health care professionals.

Function	Quotes from participants	Action taken
Structure	“Some questions are truncated in the paper.” “Takes a long time to read.” “Print all the stems on the same page.” “Shorten the questionnaire by reducing the length of the items.”	Shorten the questionnaire by reducing the length of the items. Limit examples and descriptive statements in question stems. Switch to landscape format to reduce the length of the survey to three pages.
Clarity	“RCT, what does it mean? Only the abbreviation is given.” “Should have presented the answer stems in tables or using numbers instead of bullet points.” “I think only the options are quite long.” “Not sure or uncertain is the answer that I was looking for.”	Simplify the language and minimize jargon. Revise question stems to statements to reduce length and increase clarity.
Applicability	“The questionnaire is different to the context of this app.” “We usually use it when we need it, but the question is about entertainment. Not relevant.” “It is not about entertainment, it’s whether I want it or not.” “Credibility is granted by hospital. Should not doubt it.” “App store description is not usually read by anyone. Therefore it is probably not important to assess.”	Reword item stems to better highlight app functionality and relevance. Reduce descriptive statements to minimize assumptions about app use. Remove irrelevant items such as app store description. Reduce items on entertainment and gamification.
Usability	“Likert scale should be reversed, as in 5 should be extremely satisfied.” “Should have presented the answer stems in tables or using numbers instead of bullet points.” “Introduce an additional option for those who do not know the answer.”	Change the answer stems to a simple 5-point Likert scale. Add a new answer stem for “don’t know.” Simplify design and remove Likert descriptors.

Findings from the qualitative assessment of the questionnaire (ie, interviews with HCPs) shed light on several areas of the questionnaire for focused improvement: (1) shorten the questionnaire by reducing the length of the answers, (2) further simplify the language and minimize jargon, and (3) introduce an additional option for those who do not know the answer to a specific question. It is noteworthy that no new items were proposed by the HCPs. Based on the findings, we changed the question stems from question forms to statements, for example, (1) “arrangement and size of buttons, icons, and menus on the screen is appropriate” and (2) “it uses strategies to increase engagement through entertainment (e.g., strategies such as interactivity/gamification).” Moreover, we limited examples and/or descriptive statements in question stems to a minimum. Simultaneously, we changed the answer stems to a simple 5-point Likert scale without embedded descriptive statements: strongly agree, agree, neutral, disagree, and strongly disagree. Finally, we added a new answer stem: “don’t know.”

In addition, we redesigned the questionnaire by changing its orientation from portrait to landscape. The reasons for this change were to limit the length of the questionnaire to 3 pages despite including additional questions and to provide an easy-to-complete format. The final version of the questionnaire at the end of phase 1 consisted of 26 questions spread across 5 domains: engagement (6 items), functionality (5 items), aesthetics (4 items), information (7 items), and subjective quality (4 items). The length of the questionnaire was deemed appropriate by the committee (Multimedia Appendix 2).

### Phase 2: Reliability and Construct Validity of pMARS

We distributed 197 printed questionnaires among HCPs in person, and 189 participated, resulting in a 95.9% response rate. Additionally, 29 HCPs accessed the survey through the hospital intranet, bringing the total number of responses to 218. A further 15 REDCap surveys were incomplete, resulting in a REDCap complete response rate of 65.9%. We were unable to calculate an overall response rate for REDCap as we could not track the reach through the hospital intranet. Among the in-person and intranet-administered questionnaires returned, the proportion of nonresponses per question item ranged from 0% to 13.3%, with the item “Would you pay for this app?” showing the highest nonresponse rate (n=29). Most items (29/30; 96.7%) had less than 5.1% missing responses. Detailed item-level missing data, including counts of “don’t know” responses, are provided in Multimedia Appendix 3.

**Figure 3.**
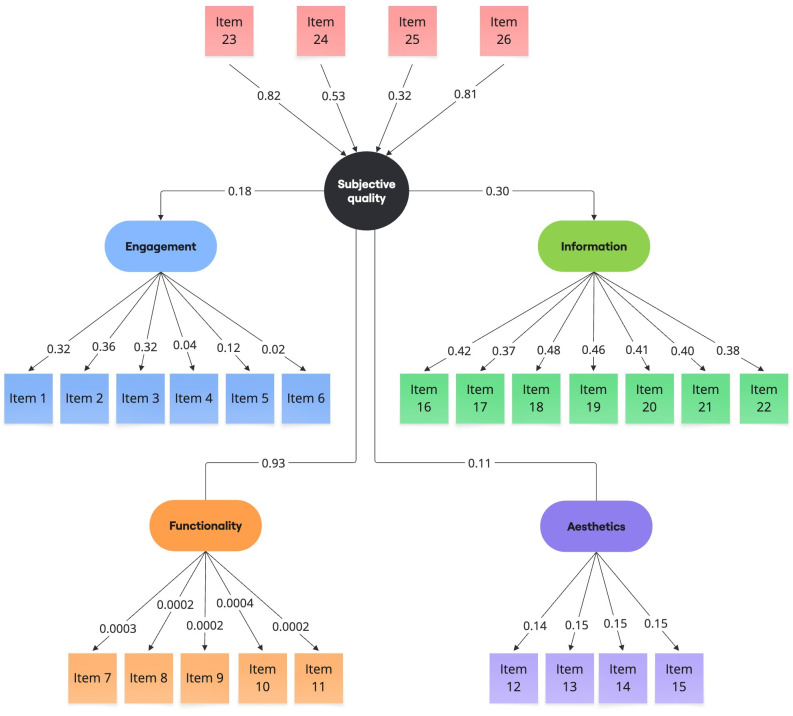
Results of the structural equation modeling.

The Cronbach α (ranging from 0.855 to 0.931) showed that the internal consistency reliability of pMARS was good to excellent for all the domains (Table 3). SEM analysis demonstrated that functionality (*P*<.001) had the strongest influence on end-user willingness to use or recommend and purchase the MHA, after adjusting for age, sex, and the HCPs’ role in the hospital (Figure 3).

For domains with Cronbach α values >0.90, we conducted a manual item review to assess potential redundancy. We examined item wording and conceptual distinctness within each domain. The review confirmed that, although some items were related, they measured complementary aspects of the construct and contributed meaningfully to the overall scale. All such items were therefore retained in the current version of pMARS.

**Table 3.** Cronbach α results for the Mobile App Rating Scale for health care professionals.

Domain and item	Scale mean if item deleted	Scale variance if item deleted	Corrected item–total correlation	Cronbach α if item deleted
Engagement: Cronbach α=0.855, number of items=6				
Item 1	17.936	8.080	0.671	0.825
Item 2	18.005	8.163	0.554	0.849
Item 3	17.675	7.597	0.721	0.815
Item 4	17.700	8.152	0.698	0.821
Item 5	17.778	8.292	0.597	0.839
Item 6	17.409	8.382	0.626	0.833
Functionality: Cronbach α=0.931, number of items=5				
Item 7	15.212	6.296	0.785	0.922
Item 8	15.212	6.059	0.864	0.906
Item 9	15.232	6.535	0.784	0.921
Item 10	15.227	6.493	0.847	0.910
Item 11	15.217	6.636	0.816	0.916
aesthetics: Cronbach α=0.928, number of items=4				
Item 12	11.296	3.576	0.813	0.912
Item 13	11.379	3.415	0.835	0.905
Item 14	11.271	3.406	0.892	0.886
Item 15	11.232	3.694	0.789	0.920
Information: Cronbach α=0.905, number of items=7				
Item 16	22.688	12.056	0.740	0.889
Item 17	22.752	12.048	0.663	0.897
Item 18	22.673	11.594	0.825	0.879
Item 19	22.713	11.569	0.811	0.881
Item 20	22.757	11.568	0.720	0.891
Item 21	22.748	11.672	0.783	0.884
Item 22	22.965	12.302	0.523	0.915

IRT (Table 4) identified that for the engagement domain, items 4 (customization) and 5 (interactivity) had a weak impact on latent constructs, whereas items 1 and 2 (both entertainment) had a higher impact on the domain. Similarly, items 9 (ease of use) and 11 (gestural design) had a weak impact on functionality, item 13 (arrangement and size of content) had a strong impact on aesthetics, and items 18 (quantity) and 19 (quality) had a strong impact on information. The engagement domain demonstrated the lowest reliability, and IRT highlighted weakly impacting items within the domain, enabling the researchers to shorten the questionnaire if required.

**Table 4.** Results of the item response theory analysis.

Domain and item	Impact^a^
Engagement	
Item 1	12.8
Item 2	5.4
Item 3	5.5
Item 4	2.9
Item 5	2.4
Item 6	5.3
Functionality	
Item 7	11.0
Item 8	41.6
Item 9	4.1
Item 10	10.8
Item 11	4.9
Aesthetics	
Item 12	12.3
Item 13	63.0
Item 14	16.6
Item 15	10.7
Information	
Item 16	3.3
Item 17	3.0
Item 18	6.8
Item 19	8.6
Item 20	3.2
Item 21	4.1
Item 22	4.2

a Overall: engagement=4.0, functionality=11.3, aesthetics=24.5, and information=4.0.

## Discussion

### Principal Findings

Our study shows that the newly developed pMARS tool has strong internal consistency reliability (Cronbach ɑ) across all domains, indicating its potential utility in assessing the quality of MHAs designed for HCPs from their perspective. Although most items performed well, item 22 of the information domain (“the app has been trialled/tested; verified by evidence”) demonstrated weak correlations (Table 3). The item was retained in the final version of pMARS as it reflects a critical aspect of evidence-based practice in clinical settings and warrants further examination in future validation studies. Furthermore, SEM generated valuable insights into interrelationships between the latent constructs. Not all items were equally good indicators for the dimensions. The strongest influence was observed between subjective quality and functionality. Similarly, items 1 to 3 of the engagement domain, items 16 to 22 of the information domain, and items 23 and 26 of the subjective quality domain demonstrated relatively strong influence.

On the other hand, IRT revealed weakly impacting items such as items 4 and 5 of the engagement domain; items 9 and 11 of the functionality domain; and items 16, 17, and 20 of the information domain. These items provide an opportunity to further shorten the questionnaire. However, it should be evaluated within the theoretical factor model of the scale, and therefore, this is an area for future research. Overall, our findings suggest that pMARS is an MHA quality assessment tool of good metric quality considering the reliability and construct validity results.

### Clinical Utility and Developmental Limitations

In the busy health care settings of Singapore, engaging HCPs in discussions unrelated to direct patient care can be challenging. With its brevity and standardized format, pMARS enables rapid assessment of MHA quality from the perspective of HCPs as clinical end users. Unlike MARS, which was designed for expert evaluators to classify and assess general MHA quality, and uMARS, which targets general end users, pMARS fills a critical gap by capturing HCP-specific clinical usability and professional decision-making considerations that are not specifically addressed by existing tools [10,21]. The use of simplified language in pMARS ensures accessibility for HCPs without requiring specific training, including those who may be unfamiliar with MHA-related terminology. Consequently, pMARS provides a practical, contextually relevant, and adaptable tool for service providers and HCPs to evaluate the quality of MHAs within real-world clinical settings. Notably, pMARS is designed as a flexible framework that can be iteratively refined to incorporate emerging clinical-, workflow-, and patient safety–related quality considerations as digital health technologies evolve.

The direct use of MARS in developing pMARS, as described by Baptista et al [27], may eliminate certain aspects of MHAs that are important to end users. To overcome this limitation, we introduced a qualitative approach using interviews with HCPs; however, the inputs generated by this procedure were limited to a few aspects of the questionnaire, as discussed in the Results section. The qualitative component introduced changes to question stems by rewording, shortening, and simplifying them without adding or removing any items from the questionnaire. According to our observations during the interviews, the interview answers were brief and the discussions were limited, possibly as a result of time pressures.

### Regulatory Challenges

Legislative frameworks such as the Health Insurance Portability and Accountability Act (HIPAA) in the United States and the Personal Data Protection Act (PDPA) in Singapore address crucial concerns for HCPs regarding MHAs, including data privacy, security, and patient confidentiality [42,43]. These regulations set important standards for protecting patient information and ensuring the reliability of MHAs. However, the adoption of such regulations varies globally, with many countries still developing or implementing their standards. As regulatory frameworks continue to evolve, the availability of a robust tool to evaluate the quality of MHAs from the perspective of HCPs remains essential to ensure these apps meet the necessary standards. Furthermore, health care organizations can use pMARS to evaluate and select suitable MHAs that comply with professional standards.

### Considerations for the Future

As mHealth technology continues to evolve, several areas for potential improvement in future versions of the pMARS tool have emerged. First, interoperability between MHAs, health systems, and electronic health records is crucial for HCPs and health care organizations when integrating these technologies [44]. Second, evaluating the effectiveness and security of communications among HCPs, including end-to-end encryption, is important for ensuring coordinated care and protecting sensitive information [45]. Third, understanding potential patient safety concerns associated with MHAs is vital for maintaining high standards of care. Lastly, language barriers among HCPs should be addressed to enhance the app’s quality, ethical considerations, and utility. Future versions of pMARS should take these evolving quality requirements into account. Additionally, with the growing integration of AI and large language models into mHealth apps, future versions of pMARS should also account for quality requirements specific to AI and AI-generated content [46,47].

### Limitations

Our study has several methodological and participant-related limitations. There were 2 methodological limitations. First, the psychometric evaluation of pMARS was based on data from only 1 MHA. This focus may affect certain aspects of statistical analysis, particularly if the MHA had quality deficiencies in specific domains. To address this limitation, future validation studies should involve a broader range of MHAs across different medical fields. Second, the IRT depends on the accuracy of the underlying model. The utility of the IRT is contingent upon how well the model reflects the data, making further validation studies essential to evaluate the appropriateness of pMARS items.

Participant-related limitations include the potential influence of the questionnaire’s ease of administration on construct validity. The simplicity and repetitive nature of the pMARS may lead to patterned responses, highlighting the need for negatively worded questions in future studies to counteract response biases. Second, the expert panel in phase 1, comprising a physician, public health expert, and human-computer interactions specialist, did not fully represent the demographics of the study participants or potential users, potentially limiting the generalizability of the pMARS tool. Further studies are necessary to assess and validate the pMARS tool across multiple MHAs. Finally, the disproportionate number of female participants should be noted as a limitation, as it may reduce the generalizability of findings to male HCPs. However, to some degree, this distribution reflects the gender composition of the hospital workforce rather than sampling bias.

### Conclusions

To our knowledge, this study is the first to develop a tool to evaluate MHAs designed for HCPs from their perspective. Additionally, it is the first study to apply IRT to assess and identify the impact of each question on the latent constructs within MARS and its derivatives. Our findings demonstrate that pMARS is a robust and metrically sound tool for evaluating MHAs intended for HCPs. It has the potential to enable HCPs to assess and recommend high-quality MHAs. We recommend that future research on pMARS incorporate IRT to refine the questionnaire by identifying and removing less-effective items, thereby enhancing its utility and precision in assessing MHA quality.

## Supplementary material

10.2196/48828 Multimedia Appendix 1 Semistructured interview guide used in phase 1.

10.2196/48828 Multimedia Appendix 2 Final version of the Mobile App Rating Scale for health care professionals questionnaire.

10.2196/48828 Multimedia Appendix 3 Item-level missing data and “don’t know” response summary for the Mobile App Rating Scale for health care professionals questionnaire (phase 2).

## Abbreviations

HCP: health care professional
HIPAA: Health Insurance Portability and Accountability Act
IRT: item response theory
MARS: Mobile App Rating Scale
MHA: mobile health app
mHealth: mobile health
PDPA: Personal Data Protection Act
pMARS: Mobile App Rating Scale for health care professionals
SEM: structural equation modeling
uMARS: user version of the Mobile App Rating Scale
