# Supplementary material for: Mobile App Rating Scale for Health Care Professionals to Assess the Quality of mHealth Apps: Questionnaire Development and Psychometric Analysis
Source: JMIR Mhealth Uhealth. 2026 Jul 31;14:e48828. doi: 10.2196/48828 (PMC13427255; doi:10.2196/48828)
Supplement: Multimedia Appendix 1 [file mhealth-v14-e48828-s001.pdf]

Qualitative interview guide for the questionnaire feedback

Introduction

Hello, my name is [redacted]. Thank you for taking the time to participate in this brief interview. The purpose of this session is to gather your feedback on the questionnaire - specifically, its content, wording, structure, and overall usability. Your feedback will help us refine the instrument further to better capture healthcare professionals' perspectives on mobile app quality.

The interview will last approximately 30 minutes. Your participation is voluntary, and no personal identifiers will be collected. We can start the interview after signing the consent form. Do you have any questions before we begin?

Demographic details:

Gender: _____________
Age: ________________
Role in the hospital: ________________________

Section 1: Overall impressions

1. What do you think about this questionnaire overall?
    a. Prompt: How was your experience completing it? Was it clear, confusing, or too long/short?

2. Is there anything you particularly liked about the questionnaire?
    a. Prompt: For example, were there sections or questions that felt relevant or well-structured?

3. Is there anything you did not like about the questionnaire?
    a. Prompt: Were there parts, e.g., question items or sections, that felt repetitive, unclear, or unnecessary?

Section 2: Relevance and clarity of questions

4. Were there any questions or sections that you found not very useful or irrelevant?
    a. Prompt: Which ones, and why?

5. Were there any questions for which you could not find a suitable answer option?
    a. Prompt: Can you give an example where your preferred answer wasn't listed? What were your preferred response options?

6. Do you have any suggestions for improving the wording of the questions or response options?
    a. Prompt: Were there any terms that could be simplified or clarified? Any specific words or terms that could be added to the questions or responses?

Section 3: Coverage of domains

7. Did you find any important domains or topics missing from this questionnaire?
    a. Prompt: For example, were there aspects of mobile app quality or functionality you think should be included?

8. Do you have any suggestions for additional areas that should be captured in future versions?
    a. Prompt: Anything personally important to you when evaluating mobile apps?

Section 4: Length and completion time

9. You took approximately ___ minutes and ___ seconds to complete the questionnaire. How did you feel about the time required?

   a. Prompt: Was it too long, too short, or about right?

Section 5: Final reflections

10. Do you have any other comments or suggestions about the questionnaire?
    a. Prompt: Anything else you would like to share about your experience?

Close

Thank you very much for your valuable time and feedback. Your input will help us refine and improve the questionnaire for future use.
