# Supplementary material for: Mobile App Rating Scale for Health Care Professionals to Assess the Quality of mHealth Apps: Questionnaire Development and Psychometric Analysis
Source: JMIR Mhealth Uhealth. 2026 Jul 31;14:e48828. doi: 10.2196/48828 (PMC13427255; doi:10.2196/48828)
Supplement: Multimedia Appendix 2 [file mhealth-v14-e48828-s002.pdf]

Name of the App: ____ **Overall 5-star user rating (App Store):** ____________________

Developer: ______________________________ **Last update:** _______________________ **Intended audience:** ___________________________________

Operating system used for evaluation: ☐ Apple ☐ Android ☐ Other **Device:** ☐ Smart phone ☐ Tablet ☐ Other

Version used for evaluation: ☐ Free version ☐ Paid version

Cost of the paid version/subscription: _________ (Weekly / Monthly / Annually / One-time)

Password required: ☐ Yes ☐ No **Two-factor authentication:** ☐ Yes ☐ No **Other security measures:** _________________________

Compliant with hospital/institutional guidelines: ☐ Yes ☐ No ☐ Not sure **Compliant with legal regulations:** ☐ Yes ☐ No ☐ Not sure

Brief description (if any): ____

SN	Question	Choices	Remarks
1<br>Background	1.1 Have you downloaded the app?<br><br>1.2 Have you used the app at work?<br><br>1.3 How often have you used the app?<br><br>1.4 On average, how long do you use it for, each time?	O Yes<br>O No<br><br>O Yes<br>O No<br><br>O A few times a day (often)<br>O A few times a week (moderate)<br>O A few times a month (seldom)<br>O Never<br><br>O 0-5 minutes<br>O 6-10 minutes<br>O 11-15 minutes<br>O 16-20 minutes<br>O >20 minutes	

SN	Question	Strongly Disagree 1	Disagree 2	Neutral 3	Agree 4	Strongly Agree 5	Don't know
2 Engagement	2.1 **Entertainment:** The app is fun/entertaining to use.	O	O	O	O	O	O
	2.2 **Entertainment:** It uses strategies to increase engagement through entertainment (e.g., strategies such as interactivity / gamification).	O	O	O	O	O	O
	2.3 **Interest:** The app is interesting to use.	O	O	O	O	O	O
	2.4 **Customisation:** It provides and retains all necessary settings/ preferences for apps features (e.g., font type, font size, content, notifications, etc.).	O	O	O	O	O	O
	2.5 **Interactivity:** It allows user input, provides feedback on inputs, and contains prompts (e.g., sharing options, notifications, submitting feedbacks, etc.).	O	O	O	O	O	O
	2.6 **Target group:** The app content (visual information, language, design) is appropriate for my work/needs.	O	O	O	O	O	O
	Please write down your comments, if any:						
3 Functionality	3.1 **Performance:** App features (functions) and components (buttons/menus) work correctly.	O	O	O	O	O	O
	3.2 **Performance:** App features (functions) and components (buttons/menus) work quickly/efficiently.	O	O	O	O	O	O
	3.3 **Ease of use:** It is easy to learn how to use the app (intuitive), i.e., menu labels, icons, and instructions are clear.	O	O	O	O	O	O
	3.4 **Navigation:** Moving between screens is logical, appropriate, and uninterrupted.	O	O	O	O	O	O
	3.5 **Gestural design:** Interactions with the app (taps/ swipes/ pinches/ scrolls) are consistent and intuitive across all components of the app.	O	O	O	O	O	O
	Please write down your comments, if any:						
4 Aesthetics	4.1 **Layout:** Arrangement and size of buttons, icons, menus on the screen is appropriate.	O	O	O	O	O	O
	4.2 **Layout:** Arrangement and size of content on the screen is appropriate and zoomable if needed.	O	O	O	O	O	O
	4.3 **Graphics:** The quality/resolution of graphics used for buttons, icons, menus, content is good.	O	O	O	O	O	O
	4.4 **Visual appeal:** The app looks good.	O	O	O	O	O	O
	Please write down your comments, if any:						

SN	Question	**Strongly Disagree 1**	**Disagree 2**	**Neutral 3**	**Agree 4**	**Strongly Agree 5**	**Don't know**
5 Information	5.1 **Accuracy of app description:** App contains what is described in app store.	O	O	O	O	O	O
	5.2 **Goals:** App has specific, measurable and achievable goals (specified in app store description or within the app itself).	O	O	O	O	O	O
	5.3 **Quality of information:** App content is correct and well-written.	O	O	O	O	O	O
	5.4 **Quantity of information:** The extent coverage (of content) is within the scope of the app and comprehensive.	O	O	O	O	O	O
	5.5 **Visual information:** Visual explanation of concepts – through charts, graphs, images, videos, etc. – is accurate and appropriate.	O	O	O	O	O	O
	5.6 **Credibility**: The app comes from a legitimate source (specified in app store description, within the app, or known information to you).	O	O	O	O	O	O
	5.7 **Evidence base:** The app has been trialled/tested; verified by evidence (in published scientific literature).	O	O	O	O	O	O
	Please write down your comments, if any:						
6 Subjective Quality	6.1 I would recommend this app to people who might benefit from it.	O	O	O	O	O	O
	6.2 How many times do you think you would use this app in the next 12 months if it was relevant to your needs?	None O	1-2 O	3-10 O	10-50 O	>50 O	O
	6.3 Would you pay for this app?	Yes O		No O		Maybe (E.g.: Depends on the price) O	
	6.4 What is **your overall star rating** of the app? Please **circle** your answer.	1 ★	2 ★★	3 ★★★	4 ★★★★	5 ★★★★★	
	Additional Comments, if any:						
