# Supplementary material for: Mobile App Rating Scale for Health Care Professionals to Assess the Quality of mHealth Apps: Questionnaire Development and Psychometric Analysis
Source: JMIR Mhealth Uhealth. 2026 Jul 31;14:e48828. doi: 10.2196/48828 (PMC13427255; doi:10.2196/48828)
Supplement: Multimedia Appendix 3 [file mhealth-v14-e48828-s003.pdf]

Item no	Questionnaire item	No. of non-respondents	No. of "don't know" responses	Total respondents	Item-level non-response rate (%)
1	1.1 Have you downloaded NTFGH LabMed app?	0	NA	218	0.00%
2	1.2 Were you using NTFGH LabMed app at work?	4	NA	218	1.83%
3	1.3 How often do you use the NTFGH LabMed mobile app?	3	NA	218	1.38%
4	1.4 On average, how long do you use it for, each time?	8	NA	218	3.67%
5	2.1 Entertainment: The app is fun/entertaining to use.	1	13	218	0.46%
6	2.2 Entertainment: It uses strategies to increase engagement through entertainment (e.g., strategies such as interactivity / gamification).	1	15	218	0.46%
7	2.3 Interest: The app is interesting to use.	1	13	218	0.46%
8	2.4 Customization: It provides and retains all necessary settings/ preferences for apps features (e.g., font type, font size, content, notifications, etc.).	1	13	218	0.46%
9	2.5 Interactivity: It allows user input, provides feedback on inputs, and contains prompts (e.g., sharing options, notifications, submitting feedback, etc.).	1	14	218	0.46%
10	2.6 Target group: The app content (visual information, language, design) is appropriate for my work/needs.	1	12	218	0.46%
11	3.1 Performance: App features (functions) and components (buttons/menus) work correctly.	1	14	218	0.46%
12	3.2 Performance: App features (functions) and components (buttons/menus) work quickly/efficiently.	1	14	218	0.46%
13	3.3 Ease of use: It is easy to learn how to use the app (intuitive), i.e., menu labels, icons, and instructions are clear.	1	13	218	0.46%
14	3.4 Navigation: Moving between screens is logical, appropriate, and uninterrupted.	1	13	218	0.46%
15	3.5 Gestural design: Interactions with the app (taps/ swipes/ pinches/ scrolls) are consistent and intuitive across all components of the app.	1	13	218	0.46%

**16**	4.1 Layout: Arrangement and size of buttons, icons, menus on the screen is appropriate.	1	13	218	0.46%
**17**	4.2 Layout: Arrangement and size of content on the screen is appropriate and zoomable if needed.	1	13	218	0.46%
**18**	4.3 Graphics: The quality/resolution of graphics used for buttons, icons, menus, content is good.	1	13	218	0.46%
**19**	4.4 Visual appeal: The app looks good.	1	12	218	0.46%
**20**	5.1 Accuracy of app description: App contains what is described in app store.	1	13	218	0.46%
**21**	5.2 Goals: App has specific, measurable and achievable goals (specified in app store description or within the app itself).	1	14	218	0.46%
**22**	5.3 Quality of information: App content is correct and well-written.	1	13	218	0.46%
**23**	5.4 Quantity of information: The extent coverage (of content) is within the scope of the app and comprehensive.	1	13	218	0.46%
**24**	5.5 Visual information: Visual explanation of concepts – through charts, graphs, images, videos, etc. – is accurate and appropriate.	1	15	218	0.46%
**25**	5.6 Credibility: The app comes from a legitimate source (specified in app store description, within the app, or known information to you).	1	13	218	0.46%
**26**	5.7 Evidence base: The app has been trialed/tested; verified by evidence (in published scientific literature).	1	14	218	0.46%
**27**	6.1 I would recommend this app to people who might benefit from it.	4	20	218	1.83%
**28**	6.2 How many times do you think you would use this app in the next 12 months if it was relevant to your needs?	10	14	218	4.59%
**29**	6.3 Would you pay for this app?	29	0	218	13.30%
**30**	6.4 What is your overall star rating of the app? Please circle your answer.	11	2	218	5.05%
